# Recovery of High Quality Metagenome-Assembled Genomes From Full-Scale Activated Sludge Microbial Communities in a Tropical Climate Using Longitudinal Metagenome Sampling

**DOI:** 10.3389/fmicb.2022.869135

**Published:** 2022-06-13

**Authors:** Mindia A. S. Haryono, Ying Yu Law, Krithika Arumugam, Larry C. -W. Liew, Thi Quynh Ngoc Nguyen, Daniela I. Drautz-Moses, Stephan C. Schuster, Stefan Wuertz, Rohan B. H. Williams

**Affiliations:** ^1^Singapore Centre for Environmental Life Sciences Engineering, National University of Singapore, Singapore, Singapore; ^2^Singapore Centre for Environmental Life Sciences Engineering, Nanyang Technological University, Singapore, Singapore; ^3^School of Biological Sciences, Nanyang Technological University, Singapore, Singapore; ^4^School of Civil and Environmental Engineering, Nanyang Technological University, Singapore, Singapore

**Keywords:** genome-resolved metagenomics, environmental microbiomes, wastewater treatment, metagenome binning, time series, metagenomics, activated sludge, metagenome assembly

## Abstract

The analysis of metagenome data based on the recovery of draft genomes (so called metagenome-assembled genomes, or MAG) has assumed an increasingly central role in microbiome research in recent years. Microbial communities underpinning the operation of wastewater treatment plants are particularly challenging targets for MAG analysis due to their high ecological complexity, and remain important, albeit understudied, microbial communities that play ssa key role in mediating interactions between human and natural ecosystems. Here we consider strategies for recovery of MAG sequence from time series metagenome surveys of full-scale activated sludge microbial communities. We generate MAG catalogs from this set of data using several different strategies, including the use of multiple individual sample assemblies, two variations on multi-sample co-assembly and a recently published MAG recovery workflow using deep learning. We obtain a total of just under 9,100 draft genomes, which collapse to around 3,100 non-redundant genomic clusters. We examine the strengths and weaknesses of these approaches in relation to MAG yield and quality, showing that co-assembly may offer advantages over single-sample assembly in the case of metagenome data obtained from closely sampled longitudinal study designs. Around 1,000 MAGs were candidates for being considered high quality, based on single-copy marker gene occurrence statistics, however only 58 MAG formally meet the MIMAG criteria for being high quality draft genomes. These findings carry broader broader implications for performing genome-resolved metagenomics on highly complex communities, the design and implementation of genome recoverability strategies, MAG decontamination and the search for better binning methodology.

## Introduction

Over the course of the last half decade the use of genome-resolved metagenome analysis has become a common approach for dealing with whole community metagenome data collected from microbiomes and complex microbial communities (Quince et al., 2017b). Starting with deeply sequenced genomic DNA, metagenome assembly is performed in order to reconstruct short fragments of the underlying member genomes, which are then analyzed further using data clustering procedures (genome binning Sangwan et al., 2016) with the objective of recovering draft genomes of the member species, referred to as metagenome-assembled genomes (MAG). This approach, now readily deployable due to the availability of near-automated bioinformatics workflows (Uritskiy et al., 2018; Kieser et al., 2020), has been successfully used on a great variety of microbial communities (Hu et al., 2016; Parks et al., 2017; Tully et al., 2018; Nayfach et al., 2019; Pasolli et al., 2019; Stewart et al., 2019; Almeida et al., 2021; Singleton et al., 2021) and has resulted in recovery of draft genomes for many new species that would have most likely remained uncharacterised due to a lack of knowledge of their required culture conditions (Parks et al., 2017).

Despite impressive accomplishments, the MAG approach still harbors many challenges and limitations. By nature, short read metagenome assemblies remain highly fractionated, resulting from the limited ability of short read sequencing to accurately capture complex repeat regions (Chen et al., 2020) and the difficulties encountered in reconstructing sequence from closely related strains or sub-species (Quince et al., 2017a, 2021; Bertrand et al., 2019; Vicedomini et al., 2021). In practice a draft genome obtained from these methods would contain at best, tens and, more typically, hundreds, of distinct contigs, and so there are inherent difficulties in accurately determining the degree of genome completeness and the extent of contamination from non-cognate genomes (Chen et al., 2020), and in identifying the presence of horizontally transferred sequence (Douglas and Langille, 2019). Another limitation relates to impact of the eco-genomic complexity of the community under study, both in terms of genomic diversity, particularly at sub-species or strain level, but also in terms of overall community richness and evenness (Quince et al., 2017b). When applied to microbial communities of high complexity, a typical MAG analysis will return many draft genomes of low quality, as defined by currently accepted criteria (Bowers et al., 2017).

Some of these challenges may be addressed using emerging methods, such as long-read sequencing (Arumugam et al., 2021; Singleton et al., 2021), synthetic long-read methods (Bishara et al., 2018) and adaptations of chromosome conformation capture methods (DeMaere and Darling, 2019; Bickhart et al., 2022). However, all of these new techniques are themselves complex and contain their own limitations, and since the vast majority of non-amplicon metagenome data has been collected using Illumina shotgun sequencing, there remains a clear need to develop more refined methods to recover genomes from short read metagenome assemblies.

Complex microbial communities associated with full-scale wastewater treatment plants (activated sludge) are particularly challenging targets for MAG-based analyses due to high species richness, high species evenness and extent of genetic diversity (Law et al., 2016; Pérez et al., 2019; Yang et al., 2020; Ye et al., 2020; Singleton et al., 2021). Recent comparative analyses undertaken with amplicon sequencing surveys suggest that these activated sludge communities are more complex than the host-associated microbiomes, including the human fecal microbiomes, by an order of magnitude (Wu et al., 2019). To date, several MAG-based analyses of activated sludge communities have been reported, varying in sequencing depth, raw sequence and availability of recovered genome (MAG) sequence, including one recently published study that employed long-read metagenomics (Singleton et al., 2021). In this paper, we consider strategies for recovery of MAG sequence from time series metagenome surveys of full-scale activated sludge microbial communities. We generate MAG catalogs from this set of data using several different strategies, including the use of multiple individual sample assemblies, two variations on multi-sample co-assembly and a recently published MAG recovery workflow using deep learning (Nissen et al., 2021). We examine the strengths and weaknesses of these approaches in relation to MAG yield and quality, and present a catalog of non-redundant draft genomes comprised of at least putatively high quality under the MIMAG criteria. All raw data and high quality MAG sequence have been made available via NCBI (BioProject Accession PRJNA731554), and key data products, including metagenome assemblies and the complete set of recovered MAG sequence data, are being made publicly available on Zenodo (doi:10.5281/zenodo.5215738).

## Results

### Summary of Data Obtained and Overall Study Design

As part of a long-term sampling project surveying the microbial ecology of wastewater treatment in tropical climates, we sampled activated sludge from aerobic-stage tanks in a full-scale wastewater treatment plant in Singapore, known to perform enhanced biological phosphorus removal (EBPR) and previously studied by us in Law et al. (2016), obtaining 24 samples over approximately a 10 month period. The median sampling interval was 7 days (mean 13 days, with range 7–56 days). At each sampling event, we obtained samples for DNA extraction from the aerobic treatment tank (including a panel of co-assayed physico-chemical measurements), and performed whole community shotgun metagenome sequencing on all samples. In total, we obtained 1.5 billion reads with a mean of 62.6 M reads per sample (range: 45.7–101.4 M; Supplementary Table 1). From these data we constructed catalogs of metagenome-assembled genomes (MAG) using several approaches as described below.

In our primary analysis, we performed both individual sample assembly of data from each of the 24 samples and co-assembly of the same ensemble of data (see Section 5.2), in order to formally compare the results of each of these two major approaches to MAG-based analysis. Metagenome assembly was performed using metaSPAdes (Nurk et al., 2017) and genome binning was performed using MetaBAT2 (Kang et al., 2019) in both cases. From the co-assembly, we generated two sets of MAGs, one using coverage profiles generated across all 24 samples and the other generated using the entire read set treated as a single meta-sample (see Section 5.2), which we refer to as multi-BAM and single-BAM co-assembly binning, respectively. As a secondary analysis, we performed metagenome binning using a recently published deep learning workflow called VAMB (Nissen et al., 2021), which is described later in the article.

### General Features of MetaBAT2-Based MAG Recovery

A total of 7,138 MAGS were recovered from the three types of assembly-binning workflows. Between 94 and 273 MAGs (mean 143) were obtained from each individual sample assembly, with a total of 3,429 MAGs being generated from all 24 individual assemblies (Table 1 and Supplementary Table 2). Approximately 10% and 27% of individual sample assembly MAGs were candidates for being high (pHQ) and medium quality (MQ) under the MIMAG criteria (Bowers et al., 2017) (see Section 5.8). The single-BAM and multi-BAM co-assembly binning workflows returned 1,997 and 1,712 MAGs, respectively (Table 1). The proportions of pHQ- and MQ-MAGs obtained from co-assemblies were higher compared to those observed from the ensemble of individual sample assemblies (Table 1), with 14.3% and 17.7% being classifiable as pHQ-MAGs in the single-BAM and multi-BAM co-assembly binning, respectively, and approximately 30% of MAG from each type of co-assembly binning workflow, holding MQ status.

**Table 1:** Number of MAGs (percentage of the total observed within workflow) from different assembly-binning workflows categorized by initial quality evaluation.

**Assembly-binning procedure**		**Putative genome quality**			

	**Total**	**High**	**Medium**	**Low**	**Unclassifed**
Individual assemblies (*n*=24)	3,429	341 (9.9%)	934 (27.2%)	1,775 (51.8%)	379 (11.1%)
Co-assembly, single-BAM	1,997	285 (14.3%)	589 (29.5%)	878 (44.0%)	245 (12.3%)
Co-assembly, multi-BAM	1,712	303 (17.7%)	532 (31.1%)	641 (37.4%)	236 (13.8%)
VAMB	1,941	156 (8.0%)	475 (24.5%)	1,293 (66.6%)	17 (0.9%)

*Percentage of total MAG number per workflow in brackets*.

The proportion of reads mapped to co-assemblies was higher (mean 92%; *n*=2) than the proportion observed to map to individual sample assemblies (mean 67%, *n*=24) (Supplementary Table 2). At the level of recovered MAG sequence, on average 27.5% (range: 21.1–37.3%) of reads were mappable to MAG recovered from single sample assemblies, whereas for MAGs recovered from single-BAM or multi-BAM co-assembly, this increased to 67.4 and 69.3%, respectively.

**Figure 1:**
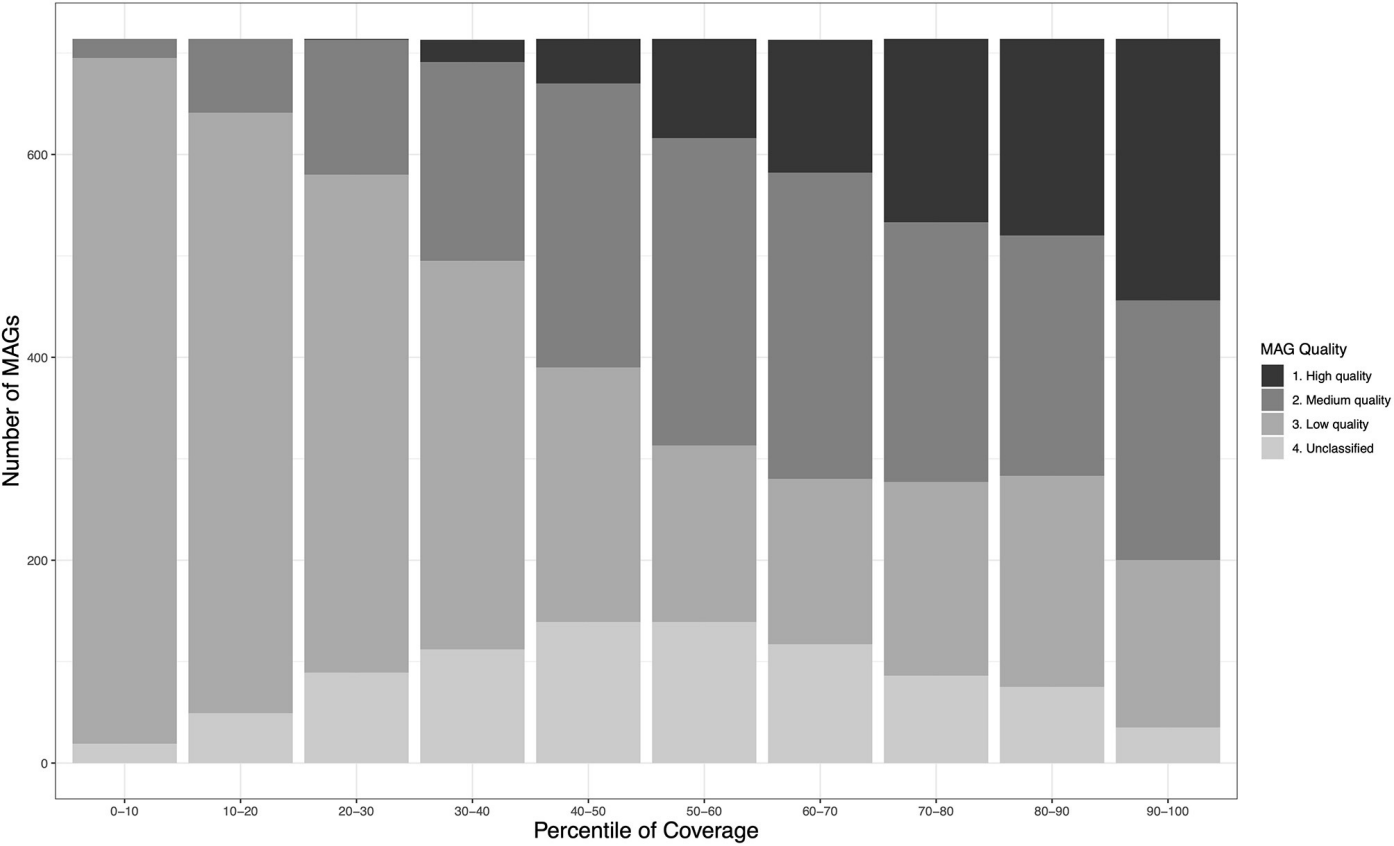
Relationship between genome quality and relative abundance within the MetaBAT2-derived MAG catalogs obtained in this study. Relative abundance is inferred using a normalised measure of coverage, permitting comparison of mean MAG coverage among the three different assembly-binning workflows (see Table 1). Within each decile of the normalised coverage distribution, the numbers of MAG meeting each of the four MIMAG-derived quality levels is shown.

As expected, estimated MAG genome quality demonstrated a strong association with relative abundance-expressed as a normalized coverage measure that permits comparisons across workflows whose variable number of input sequence reads would bias estimation (see Section 5.2)—with the proportion of pHQ MAGs being highest in the top 10%-ile of the normalized coverage, and decreasing in a roughly uniform manner thereafter (Figure 1). A similar trend was observed for MQ-MAGs, and the proportion of poor quality MAGs expanded in the bottom 50% of the normalized coverage distribution.

Given the expected high degree of genomic redundancy among the complete set of 7,138 MAGs generated from the three assembly-binning workflows employed, the entire set was de-replicated and grouped into non-redundant genome clusters [*secondary clusters* as defined by the dRep workflow (Olm et al., 2017); see Section 5.9]. In total 2,912 non-redundant clusters where obtained, comprised of between 1 and 26 MAGs (median 2; mean 2.45) (Supplementary Table 3). Of these 2,912 secondary clusters, 382 (13.1%) contained at least one MAG that was pHQ, and 690 (23.7%) contained MAGs that were MQ at best, with the remainder containing MAGs of either low quality (LQ; *n*=1,576; 54.1%) or else unclassifiable (UC; *n*= 264; 9.1%).

To provide some insight into the extent to which the recovered MAGs are representative of the underling community composition, we screened all assembled contigs for the presence of 16S SSU-rRNA genes using BAsic Rapid Ribosomal RNA Predictor (Barrnap version 0.9) [ref] and then dereplicated these sequences using CD-hit [ref] (see Section 5), under the simplifying assumption that the total number of non-redundant could be considered an rough approximation for the lower limit of overall community complexity. The number of non-redundant 16S sequences was observed to be 3,199 (from 3,489 detected in total) from the co-assembly and 5,381 from all 24 single sample assemblies (from 14,591 detected in total). Thus, we based on the set of the 2,912 secondary clusters of MAGs defined above, the assembly-binning approach is probably capturing, at most, no more than 55% ($\tilde{2}$9,12/5,381) of the recoverable taxonomic content of the community.

### Comparative Analysis of Binning Strategies Using MetaBAT2

**Figure 2:**
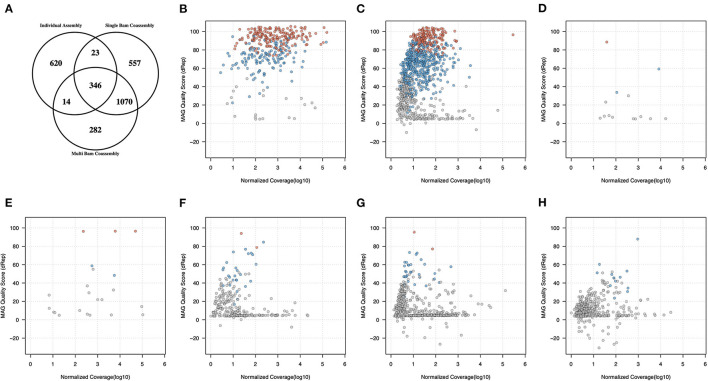
Inter-relationships between genome quality, relative abundance and recovery workflow type for 7.138 MAGs recovered from the use of MetaBAT2. **(A)** secondary cluster membership by workflow 7,138 MAG categorised into 2,912 secondary clusters; the remaining panels show the highest observed genome quality with a secondary cluster (vertical axis) against normalised coverage measure (horizontal axis) for secondary clusters **(B)** containing MAGs from all three workflows (*n*=346); **(C)** recovered from both single-BAM and multi-BAM co-assembly workflows (*n*=1070); **(D)** recovered from both individual assemblies and multi-BAM co-assembly (*n*=14); **(E)** recovered from both individual assemblies and single-BAM co-assembly (*n*= 23); **(F–H)** recovered solely from multi-BAM co-assembly (*n*= 282), single-BAM co-assembly (*n*= 557) and individual assemblies (*n*= 620), respectively. Secondary clusters containing pHQ, MQ, and LQ MAGs are coloured in red, blue, and grey, respectively.

To gain further insight into the effectiveness and inter-relationship of each type of genome recovery workflow, the set of 2,912 non-redundant clusters were further categorized according to the the types of workflow which had contributed at least one genome to a given non-redundant cluster (Supplementary Table 4 and Figure 2A). Of these 2,912 secondary clusters, 346 (11.9%) contained genomes contributed from both individual sample assemblies and both types of co-assembly binning procedure, and 1,070 (36.8%) contained MAGs recovered from both types of co-assembly but not from any individual sample assembly (Figure 2A). Relatively few MAGs were observed arising from individual sample assembly and from either, but not both, types of co-assembly (23 and 14 secondary clusters, respectively, against single-BAM and multi-BAM; multi-BAM; Figure 2A). In contrast, we observed substantial numbers of secondary clusters that were only comprised of MAGs obtained from within one of the three workflows (Figure 2A).

We then examined how these associations were patterned by genome quality and relative abundance, using a composite quality statistic as defined in the dRep pipeline and a normalized measure of MAG coverage that adjusted for differences in coverage that are present across the three types of workflows (Figures 2B–H). Each secondary clusters was represented by the best quality MAG observed in that cluster, as defined by the maximum dRep quality score within the highest MAG quality category from that cluster.

We observed that the 346 secondary clusters comprised of MAGs recovered from all three workflows had the highest overall coverage and over half of these secondary clusters contained at least one pHQ genome (189/346 or 54.6%). In the larger set of 1070 secondary clusters that arose from both types of co-assembly workflow, 185 (17.3%) and 483 (45.1%) of these held at least one genome of pHQ and MQ level, respectively. These secondary clusters were also distributed across a lower coverage range than the previous category (Figure 2C), consistent with the expectation that co-assembly procedures can recover genomes of less common taxa. Of the remaining set of 1,496 secondary clusters from the remaining five categories there were only 8 (0.54%) which held candidates for being pHQ-MAGs, with being pHQ-MAGs, with the remainder holding unremarkable or frankly poor quality (Figures 2D–H).

**Figure 3:**
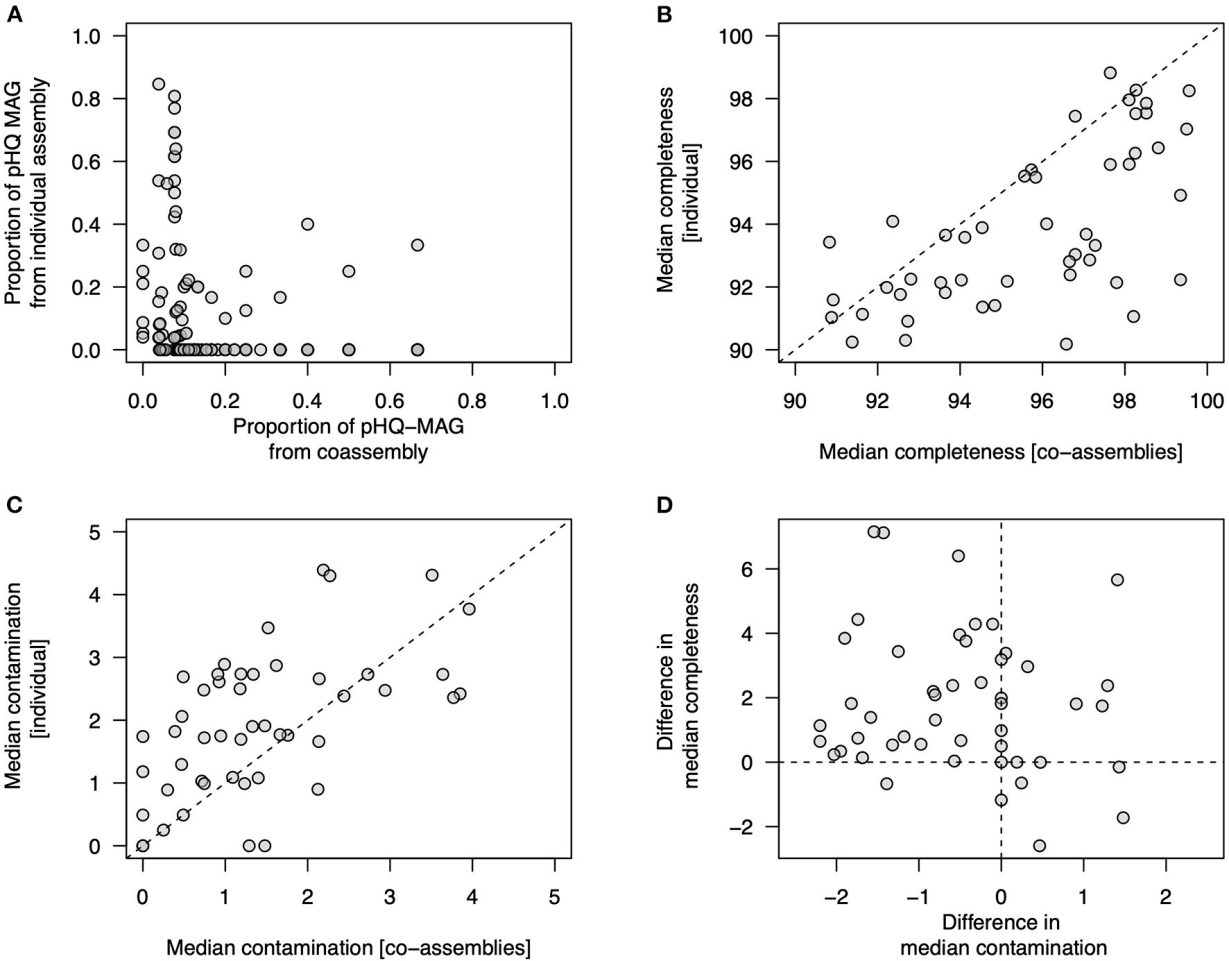
Examination of genome quality in MAGs recovered among different MetaBAT2-derived workflows. Each data point references a secondary cluster of highly related genomes obtained from the dRep workflow. **(A)** proportion of MAGs within a secondary cluster that attain pHQ quality status from co-assembly workflows (*x*-axis) and individual assemblies (*y*-axis); **(B–D)** comparison of genome quality measures from within 48 secondary clusters in which at least one pHQ MAG is observed from a co-assembly workflow and from an individual assembly; **(B)** median completeness (*C*_*p*_) observed from co-assemblies (*x*-axis) and individual assemblies (*y*-axis); **(C)** median contamination (*C*_*n*_) observed from co-assemblies (*x*-axis) and individual assemblies (*y*-axis); **(D)** relationship between the difference in median completeness observed in co-assemblies with respect to individual assemblies (*y*-axis) and difference in median contamination (co-assemblies with respect to individual assemblies) (*x*-axis).

We then undertook several secondary analyses to examine whether co-assembly or individual sample assembly showed any inherent biases in genome quality (Figure 3). Firstly, for secondary clusters that contained MAGs from all three workflows (Figures 2A,B) we examined the proportion of pHQ-MAG in secondary cluster that came from either type of co-assembly or from an individual assembly, but observed no clear pattern in relation to the origin of pHQ-MAGS (Figure 3A). Secondly, we compared completeness and contamination statistics within a subset of 48 secondary clusters that contained at least one pHQ genome sourced from co-assembly and at least one pHQ genome from an individual assembly. Removing all genomes that did not attain pHQ status, on average this subset of secondary clusters contained 1.8 pHQ-MAGs (range: 1–2) sourced from the co-assembly workflows and 6.3 pHQ-MAGs (range: 1–22) arising from the individual assembly workflow. We calculated median completeness and median contamination within each secondary cluster, conditioned on workflow type, observing a bias toward higher completeness (Figures 3B,D) and a lower contamination (Figures 3C,D) in co-assembled genomes relative to genomes obtained from individual assemblies.

Collectively, these data suggest that if we focus attention on recovered genomes that are plausibly of high quality, then these results indicate, in the communities studied here, that co-assembly conveys advantages in regards to MAG yield.

### Decontamination of Recovered Draft Genomes

To improve the number of high quality MAGs produced from the workflows above, we applied RefineM (Parks et al., 2017) to all MAGs from the three assembly procedures that possessed completeness of more than 90% (1,307 MAGs, contributed from 550 secondary clusters) regardless of their contamination and strain heterogeneity levels, as calculated by CheckM (Parks et al., 2015), considering these suitable candidates for refinement to high quality.

The results of the decontamination analysis are summarized in Table 2A. Of the 1,307 MAGs, 929 (71.1%) were classified as pHQ prior to decontamination, and of these, 855 (92.0%) retained the same quality level after application of RefineM. Of the remaining 74 pHQ-MAGs, the majority 94.6% were converted to MQ, with only four being reduced to LQ status. Of the 127 MAGs originally classified as MQ, 35.4% (45/127) attained pHQ status following application of RefineM. In the set of 251 MAGs that held UC status before decontamination, only 7.2% and 16.7% improved their quality to pHQ and MQ, respectively, suggesting that most MAGs that hold contamination above 10% are likely to be of highly flawed construction.

**Table 2:** Number of MAGs categorized by genome quality assignments before and after decontamination with RefineM for **(A)** MAGs obtained from MetaBAT2 workflows and **(B)** MAGs from VAMB workflow.

		**After**						**After**			
		**pHQ**	**MQ**	**LQ**	**UC**			**pHQ**	**MQ**	**LQ**	**UC**
	pHQ	855	70	4	0		pHQ	100	54	2	0
Before	MQ	45	80	2	0	Before	MQ	3	12	0	0
	UC	18	42	2	189		UC	1	1	0	2
	**(a) MetaBAT2 workflows**						**(b) VAMB workflow**				

*Input MAGs held CheckM-estimated completeness>90%*.

Across all MAGs, the average number of contigs removed by RefineM was 59 (range: 0–3,309) with CheckM completeness, contamination, and strain heterogeneity reduced on average by 1.7%, 5.8%, and 0.5, respectively (Supplementary Table 5).

### Genomes Recovered Using a Deep Variational Autoencoder Workflow

As a secondary, complementary analysis to the canonical approach taken above, we performed genome recovery using a recently described workflow called VAMB that utilizes deep variational autoencoders (Nissen et al., 2021). Using data from the 24 individual-sample assemblies, VAMB generated 1,941 MAGs of minimum total sequence length of 200 kbp (to match that used by the default MetaBAT2-based workflows).

**Figure 4:**
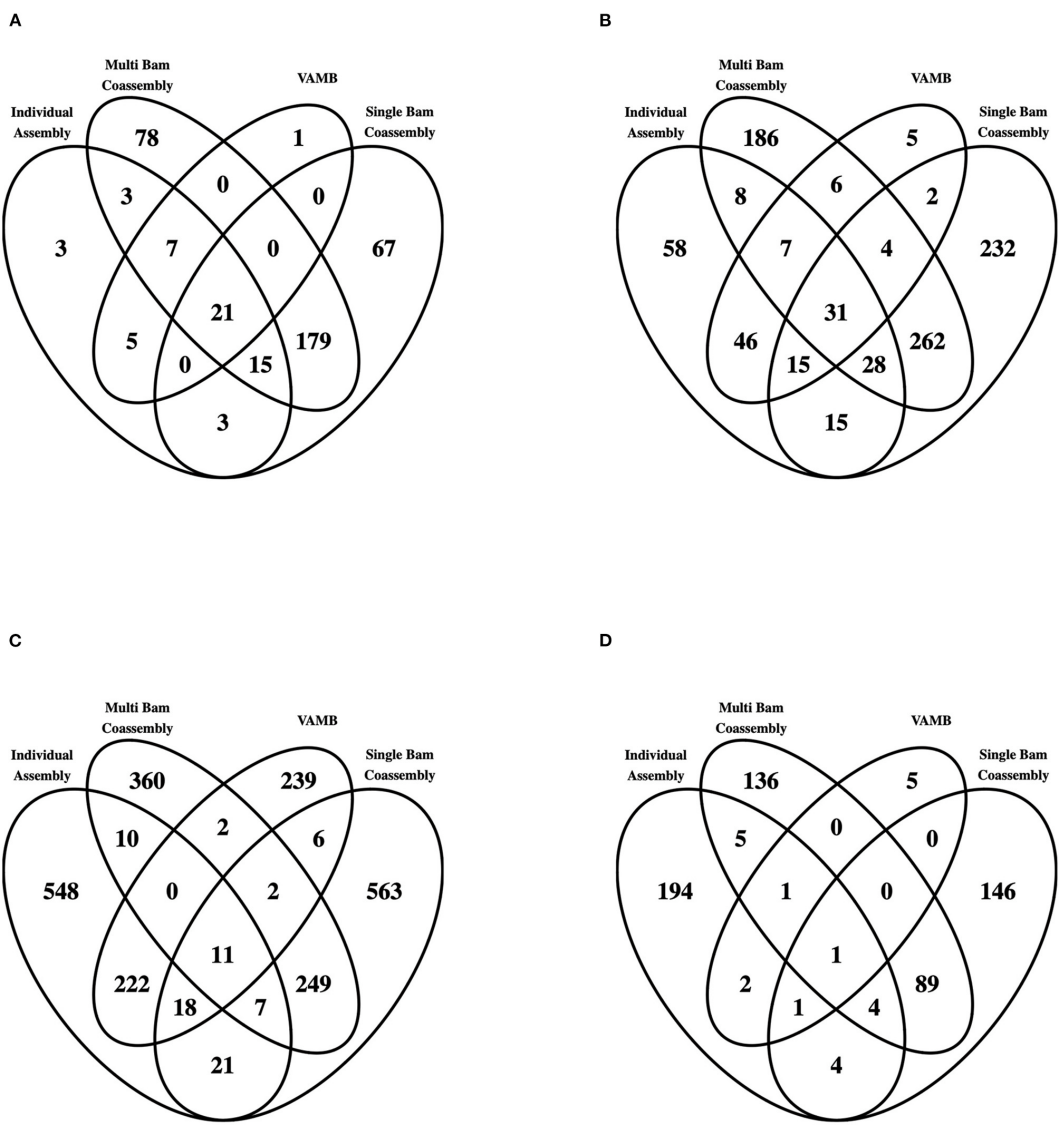
MAG recovery across all four workflows employed in this study, conditioned by genome quality. The entire set of 9,709 MAGs was categorised by genome quality level, and within each quality genome level further categorised by the workflow of origin observed with each secondary cluster. Venn diagrams showing interrelationships are shown here for **(A)** pHQ (putative high quality); **(B)** MQ (medium quality); **(C)** LQ (low quality) and **(D)** UC (unclassified).

Of the recovered draft genomes, 8.0, 24.5, 66.6, and 0.9% were classified as pHQ, MQ, LQ and UC, respectively (Table 1 and Supplementary Table 6). The pHQ-MAGs from VAMB were strongly associated with those detected by the MetaBAT2 workflows, with only 1 and 5 secondary clusters containing pHQ–MAGs (Figure 4A) and MQ-MAGs (Figure 4B), respectively, that were not recovered by any other workflow. While a substantive number of secondary clusters containing LQ-MAGs were recovered by VAMB (Table 1 and Figure 4C), interestingly, the number of secondary clusters containing UC-MAGs was two orders of magnitude lower than the number observed in the MetaBAT2 workflows (Figure 4D). This suggests that the VAMB methodology may likely provide superior control of gross contamination, although possibly at the expense of recovery of more complete, higher quality MAGs.

As above, we applied RefineM workflow to the 175 MAGs with completeness above 90% (contributed from 36 distinct secondary clusters), which were primarily comprised of pHQ-MAGs (*n*= 156, 89.14%). After application of the RefineM workflow, 59% of pHQ-MAGs retained their quality status, and 38% were reduced to MQ-status. The numbers of MAGs in remaining categories was low (Table 2B). The average number of contigs removed by RefineM was 53 (range: 0–495), and completeness, contamination and strain heterogeneity were reduced on average by 4.6%, 1.4% and 1.3 units, respectively (Supplementary Table 6).

### Catalog of High Quality Genomes From Tropical Climate Activated Sludge

The entire set of MAGs recovered from all four sources were combined into a single set of 9,079 bins (7,138 bins from the MetaBAT2 workflows and 1,941 from VAMB; see Supplementary Table 7), corresponding to 3,113 secondary clusters, as defined by dRep. Of the 9,079 MAGs defined by this analysis, 1,085 (11.9%) were categorized as pHQ. Of these 1,044 (96.2%), 124 and 5 were comprised of less than 500, 50, and 10 contigs or less, 1066 MAGs (98.3%) held an N50 of at least 10 kb and 142 MAGs (13.1%) contained at least one copy of the 5, 16, and 23S SSU-rRNA genes. The 1,085 pHQ MAGs were split among 382 different secondary clusters.

**Figure 5:**
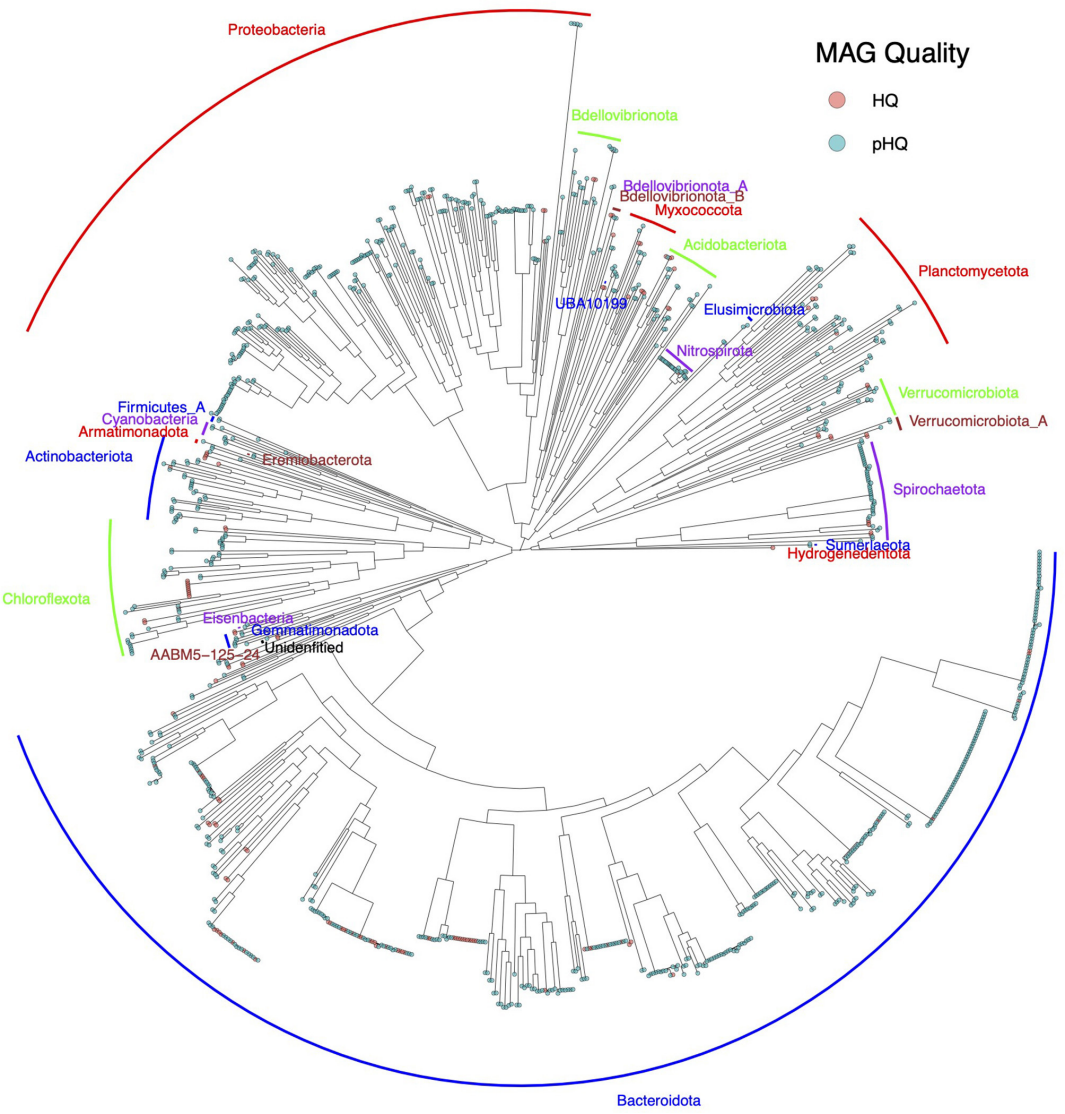
Phylogram of pHQ MAGs recovered from all four workflows used in this study. Phylum level annotations are listed as text labels. MAG holding pHQ status are highlighted in blue-green and the subset of those that hold HQ status under the MIMAG are highlighted in light red.

Taxonomic analysis (Figure 5) showed a predominance of phyla *Bacteroidota* and *Proteobacteria*, which accounted for 44.4% (482/1085 MAGs within 100 secondary clusters) and 20.6% (223/1,085 MAGs in 98 secondary clusters) of MAGs classified as pHQ. Other phyla that were observed at relative frequencies above 1% were *Chloroflexota* (5.4%, 59 MAGs within 22 secondary clusters), *Planctomycetota* (5.3%, 57 MAGs within 37 secondary clusters), *Spirochaetota* (4.4%, 48 MAGs within 7 secondary clusters), *Actinobacteriota* (3.8%, 41 MAGs within 19 secondary clusters), *Acidobacteriota* (2.7%, 29 MAGs within 17 secondary clusters), *Myxococcota* (2.3%, 25 MAGs within 17 secondary clusters), *Nitrospirota* (2.3%, 25 MAGs within 4 secondary clusters), *Bdellovibrionota* (2.7%, 29 MAGs within 18 secondary clusters) and *Verrucomicrobiota* (2.3%, 25 MAGs within 16 secondary clusters).

Across the 382 secondary clusters, only 14 (3.7%) were comprised of MAGs annotated to species level and 155 (40.6%) were annotated to genus level, highlighting that over half the recovered pHQ MAGs were likely to be previously uncharacterised. Species-level annotations were observed for the polyphosphate accumulating organisms (PAO) *Candidatus* Accumulibacter SK-02 (*n*= 2 MAGs) (Skennerton et al., 2015) and the cyanobacteria *Obscuribacter phosphatis* (Soo et al., 2014; Stokholm-Bjerregaard et al., 2017) (*n*= 2 MAGs), and the glycogen accumulating organism, *Candidatus* Competibacter (McIlroy et al., 2014) (*n* = 1 MAG). Interestingly, we recovered a single MAG from *Romboutsia timonensis*, a member of the human gut microbiome (Ricaboni et al., 2016), and to our knowledge not previously identified in activated sludge communities, and genomes of the methane-oxidizing bacteria *Methylosarcina fibrata* (Hamilton et al., 2015) (*n*=2 MAG). Genomes from the denitrifier *Hyphomicrobium denitrificans* (Martineau et al., 2014) were recovered (*n*=2 MAG), along with genomes from two species within the UBA2359 lineage within order *Chitinophagales* (GTDB), namely *Sphingobacteriales* bacterium TSM_CSS and *Sphingobacteriales* bacterium TSM_CSM and genomes from recently identified novel lineages in phyla *Bacteroidetes, Chloroflexi* and *Chlorobi* (see Supplementary Table 8 for full details of species-level identifications).

The ammonia-oxidizing bacteria (AOB), *Nitrosomonas* (Kowalchuk and Stephen, 2001) (*n* = 16 MAG), and the nitrite oxidizing bacteria (NOB), *Nitrospira* (Vijayan et al., 2021) (*n*=25 MAG), both key functional species in activated sludge-mediated bioprocesses, where only represented at genus level.

When we applied the more stringent form of the MIMAG criteria for high quality genomes (Bowers et al., 2017), that is, those with at least 18 tRNAs and the presence of a complete set of rRNA genes, only 58 HQ-MAGs were identified. In addition, 6 more high quality MAGs were recovered from RefineM pipeline, resulting in a total 64 high quality MAGs submitted to NCBI.

### Comparison to Other MAG Catalogs Recovered From Activated Sludge

**Figure 6:**
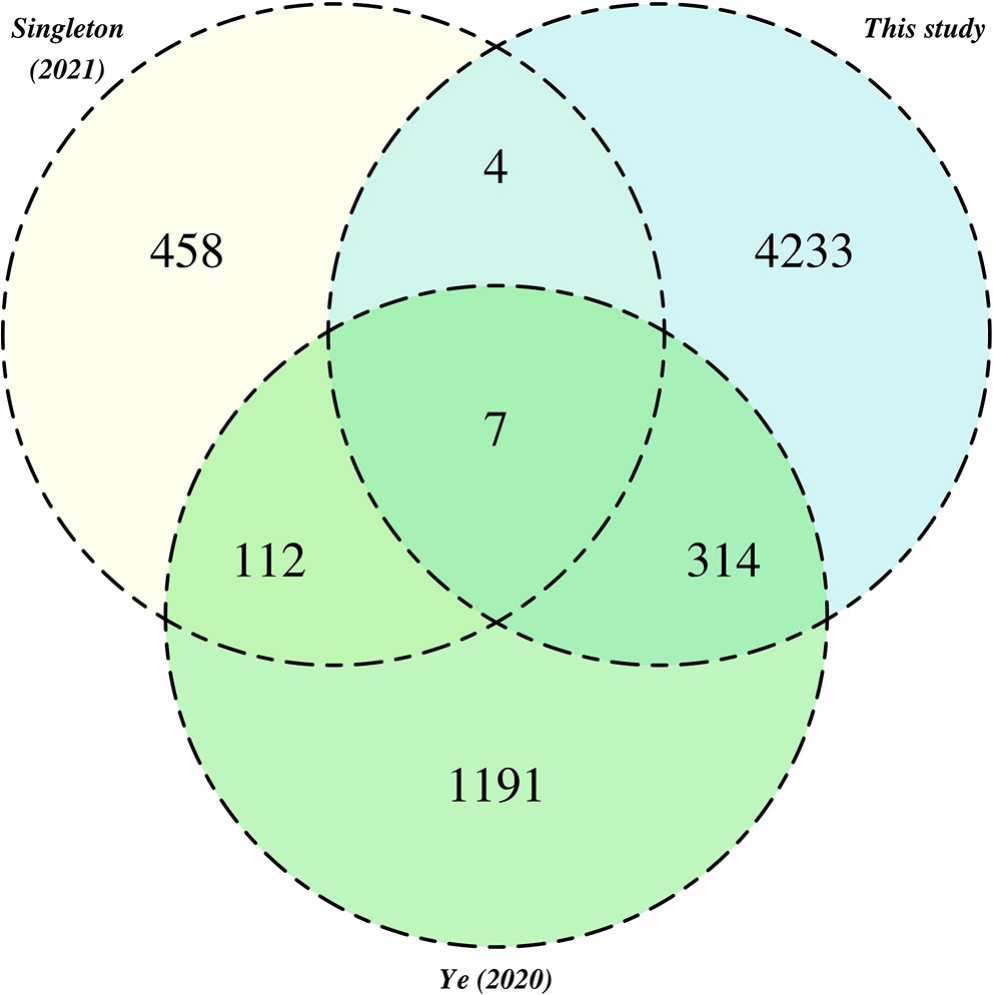
Relationships between pHQ-MAGs recovered in this study and MAGs from extant activated sludge catalogs. Counts reference secondary clusters categorised by the presence of MAGs originating from one of the three MAG catalogs.

We systematically compared our MAG catalog to several others that have been recently obtained (Ye et al., 2020; Singleton et al., 2021), using genome de-replication (see Section 5.9) and same criteria recently used in a comparative analysis of MAG catalogs from multiple cow rumen microbiomes (Watson, 2021). Collectively, this analysis defined a total of 6,328 secondary clusters, containing on average 1.9 MAGs (median 1.0; range 1–54 MAG). We examined the membership of these secondary clusters in relation to catalog of origin (Figure 6). Only 7 secondary clusters contained genomes from all three source catalogs. A larger proportion of related genomes (*n*= 314) was observed between our catalog and that of Ye et al. (2020), than between our study and the catalog of Singleton et al. (2021), which may reflect the more diverse geographies and mixture of operational regimes incorporated in the former study. We highlight however, that our analysis is retrospective and thus should be interpreted with caution.

## Discussion

In this paper we undertake a comprehensive genome-resolved metagenome survey of an activated sludge microbial community from a full-scale, tropical climate wastewater treatment plant, based on a time-series survey design. We obtain a total of just under 9,100 draft genomes, which collapse to around 3,100 non-redundant genomic clusters (defined under a stringent degree of relatedness), and we estimate the latter to capture, ar most, just over 50% of the overall community composition. Around 1000 MAGs were candidates for being considered high quality, based on single-copy marker statistics (referred to pHQ in our analysis) but 58 MAGs formally meet the MIMAG criteria for being high quality draft genomes. In building these MAG catalogs, we undertake a systematic comparison of MAG recovery strategies, based on the use of individual-sample assemblies and two variations on the use of co-assemblies (using the combination of metaSPAdes for performing assemblies and MetaBAT2 for genome recovery). Additionally, we compared these results to those obtained from the use of a recently released deep learning variational autoencoder called VAMB (Nissen et al., 2021), which appears to convey some advantages in relation to control of MAG contamination. As discussed below, these findings carry broader implications for conducting genome-resolved metagenomics on highly complex communities.

The genomes recovered at pHQ level in this study represented 11 phyla, captured at a relative frequency of above 1%, with just under half being members of *Bacteriodota* and *Proteobacteria*, and represent the most comprehensive catalog obtained from tropical climate activated sludge communities, building on our previous efforts (Law et al., 2016; Arumugam et al., 2019, 2021; Qiu et al., 2020). Wastewater microbial communities from tropical climates are understudied relative to their temperate climate counterparts, as are the bioprocesses that they support. Given the urgent need to understand the impact of climate change at microbial scales of life (Cavicchioli et al., 2019), such communities will become an increasingly important target of study, given their role as mediators of the interface between human and natural ecosystems (McLellan et al., 2015). In the present case, we obtained pHQ or confirmed HQ MAGs for expected taxa conveying key functionality to activated sludge bioprocesses including the AOB *Nitrosomonas*, NOB *Nitrospira*, the PAO *Candidatus* Accumulibacter and the GAO *Candidatus* Competibacter. Notable unexpected findings included, but were not limited to, the cyanobacterial PAO species *Obscuribacter phosphatis* and *Romboutsia timonensis*, a microbe previously identified in the human gut and plausibly an immigrant species from that source.

The large proportion of recovered genomes that hold unremarkable quality is expected, given the recognized challenges of performing these analysis on highly complex microbial communities (Pasolli et al., 2019) and the known complexity of full-scale activated sludge microbial communities, which are estimated to be more complex than the human gut microbiome by around an order of magnitude (Wu et al., 2019). The complexity of the community in regards to taxonomic novelty is also seen in the fact that around 60% of the recovered pHQ hold no assignment at species or genus level, and by the relatively low degree of recapitulation of genomes from other activated sludge catalogs. Consistent with the recognized limitations of MAG analyses conducted from short-read sequence data, the recovered genomes are unlikely to be resolved to strain level, and the size and complexity of the dataset limited the use of a recent genome-bin workflow for strain deconvolution (Quince et al., 2021) (data not shown). Nonetheless, the kind of densely sampled longitudinal data collected here is ideally suited for developing such strain-aware genome recovery methods.

As part of this analysis, we have undertaken a comprehensive comparison of individual sample assembly and co-assembly approaches for genome recovery, which has been relatively unexplored in the literature to date. Current thinking on MAG analysis suggests that assembling data from individual samples will aid the recovery of higher quality, relatively abundant genomes, while co-assembly will assist in the recovery of lower abundance genomes with the trade-off of artifacts associated with multi-sample analysis (Pasolli et al., 2019; Hofmeyr et al., 2020), including cross-sample chimeras (Chen et al., 2020), split-bins (Arumugam et al., 2021) and increased probability of recovering pan-genomic level (Chen et al., 2020), although this will no doubt be dependent on, the nature of the co-assembled samples (longitudinal vs. cross-sectional; Pasolli et al., 2019), sample replication number, genetic diversity, community complexity and, of course, sequencing depth. In their comparative analysis on co-assembly and individual assembly of infant and maternal gut microbiomes, Pasolli et al. (2019) found little difference in number or quality of recovered genomes from either method, which included an analysis of both longitudinal and cross-sectional sampling designs, concluding that application to longer time-series would likely result in higher MAG yields. The findings of the present study are consistent with that view, with substantially higher numbers of pHQ-level MAGs being recovered from co-assembly procedures. We find some clear indications that co-assembly is advantageous in regards to genome quality, and, at least in the subset of MAGs that are recovered at pHQ level by both approaches, there is clear evidence that co-assembly will provide cognate MAGs with higher completeness and lower contamination statistics, as defined by single copy marker gene analysis: the extent to which this is generalisable to other settings is unclear.

Unexpectedly, we find the two specific modes of co-assembly are each capable of high MAG yields suggesting that greater depth *per se*, as implemented in the single-BAM approach, will recover almost as many pHQ MAGs (285 vs. 303; Table 1) as the canonical differential coverage approach (multi-BAM). We would caution however, that the performance of the single-BAM co-assembly workflow may be a consequence of the high degree of relatedness between the longitudinally studied samples used in this study. In a setting where this is not the case, the rate of occurrence of artifactual and/or chimeric MAGs formation could be potentially far higher, and we would not recommend its routine application for this reason. Additionally, the computational overheads of co-assembly can be substantial, as seen in the present case, and may be untenable or impractical in some settings. Obviously this would also influence the choice of metagenome assembler, for example, MEGAHIT may be a more suitable choice of assembler than metaSPAdes for datasets at, or above, the scale of data employed here. Interestingly, as applied to this dataset, the deep-learning based VAMB workflow recovered pHQ MAGs that largely recapitulated those from the MetaBAT2 workflows. Collectively, these findings reinforce the view that MAG recovery is highly context-specific in relation to the community under study (Vollmers et al., 2017).

There remains an urgent need for methods to identify non-cognate contigs in fractionated assemblies, with the impact of contamination on gene-level becoming more widely recognized (Arkhipova, 2020), and one recently published analysis suggests that up to 15–30% of publicly-available MAGs classified at pHQ level will harbor chimeric content (Orakov et al., 2021). In the present study, we have examined removal of possible contamination using the RefineM workflow (Parks et al., 2017). Our results shed light on the strengths and weaknesses of the different recovery workflows we employed. From the MetaBAT2 workflows, there was a high degree of robustness in the case of recovered genomes that were classified at pHQ level, with over 90% retaining their pHQ status upon the application of RefineM. In the case of draft genomes that held high levels of contamination upon a backbone of high completeness, most also remained within the same genome quality category following de-contamination, suggesting that these recovered sequence constructions are fundamentally flawed. In the case of the bins recovered from VAMB, while around one third of pHQ changed quality level to MQ, there was an under-representation of complete genomes initially showing high degrees of contamination, suggesting that VAMB may be quite robust to the formation of chimeras. Whether this is a general property, or a consequence of the high redundant nature of time-series data, is a subject for further study.

Collectively, our results reinforce the ongoing need for analysis procedures suitable for recovering high quality MAGs from metagenome data, also highlighted by recent calls for more careful manual curation of recovered genomes (Chen et al., 2020; Lui et al., 2021) and the use of complementary sequencing, including long read (Arumugam et al., 2021; Singleton et al., 2021), synthetic long read (Bishara et al., 2018) and chromosome confirmation capture methods (DeMaere and Darling, 2019; Bickhart et al., 2022). Another relevant development is the direct use of assembly graphs in MAG recovery, including for the recovery of strain level sequence (Brown et al., 2020; Mallawaarachchi et al., 2020; Quince et al., 2021). Further attention could also be placed on the use of alternative feature representations for contig sequence and/or coverage data: most methods developed to date have used Euclidean space (of various dimensionality, ranging from two to several hundred), but other representations may hold substantive advantages, for example hyperspherical or hyperbolic embeddings (Ding and Regev, 2021) or related manifold learning methods e.g., as implicit in the use of VAMB (Nissen et al., 2021).

## Conclusions

Using a time-series metagenome survey of activated sludge microbial communities, we evaluate several extant strategies for obtaining MAG catalogs, showing that co-assembly offers clear advantages over single-sample assembly. We obtain a total of just under 9,100 draft genomes, which collapse to around 3,100 non-redundant genomic clusters. One thousand MAGs were candidates for being considered high quality, based on single-copy marker gene occurrence statistics, however only 58 MAG formally meet the MIMAG criteria for being high quality draft genome. More broadly, our findings have a number of broader implications in regards performing genome-resolved metagenomics on highly complex communities, the design and implementation of genome recoverability strategies, MAG decontamination and the search for better binning methodology.

## Methods

### Metagenome Extraction and Sequencing

The field sampling methodology, sample handling, DNA extraction and sequencing methods have been previously described by us Law et al. (2016). At a full-scale operational wastewater treatment plant in Singapore, treating mostly waste of domestic origin, we sampled the aerobic stage of an activated sludge tank known to perform enhanced biological phosphate removal (EBPR). At each sampling event, we obtained multiple samples for DNA extraction from the aerobic treatment tank and collected a panel of relevant physico-chemical measurements (data not analyzed in this paper). Samples were snap frozen in a liquid nitrogen dry shipper immediately upon retrieval from the tank, and transported to the laboratory for subsequent genomic DNA extraction and sequencing on Illumina HiSeq2500 using a read length of 251 bp (paired end) (see Law et al., 2016 for details of all gDNA extraction, library preparation and sequencing protocols).

### Genome-Resolved Metagenome Analysis

Unless otherwise stated data analysis was performed in the R Statistical Computing Environment (version 4.0.5) (R Core Team, 2021).

#### Initial Data Processing

The raw FASTQ files were processed using cutadapt (version 1.5, with default arguments except –overlap 10 -m 30 -q 20) (Martin, 2011).

#### Genome Recovery From Individual Sample Assemblies

From the processed read data, we initially performed individual sample assemblies using SPAdes (Nurk et al., 2017) in -meta mode with maximum *k*-mer value of 127, and performed metagenome binning using MetaBAT2 version 2.12.1 (default settings) to obtain an initial set of MAGs from each sample. Coverage for each contigs were extracted from SPAdes *k*-mer coverage, converted to log scale, and averaged per bin. To compare estimates of MAG coverage between samples, we normalized MAG coverages by centering using the per-sample mean per-MAG coverage, scaling by the per-sample standard deviation of coverage and then placing back on a positive scale by subtracting the smallest normalized coverage value across the entire set of MAGs.

#### Genome Recovery From Co-assemblies

Processed read data from the 24 samples were co-assembled with SPAdes-3.13.0 (Nurk et al., 2017) (default parameters except –meta -m 2,900 -k 21, 33, 55, 77, 99, 127 -t 50). Binning was performed on contigs over 2,500 bp in length with MetaBAT2 (Kang et al., 2019) (version 2.12.1 with default parameters except -d -t 40 -m 2,500 -v), employing two different approaches, namely: 1) using contigs from the co-assembly and 24 sorted. bam files made by aligning reads from each of the 24 datasets to the contigs from co-assembly, referred to as the *multi-BAM co-assembly* and 2) using contigs from the co-assembly and a single sorted bam file made by aligning all reads from the 24 datasets to the contigs from co-assembly (referred to as a *single-BAM co-assembly*).

#### Genome Recovery Using Deep Variational Autoencoder

The recently published metagenome binner VAMB was employed on the 24 individual sample assemblies, following the described procedure in Nissen et al. (2021). Briefly, all assembled contigs with minimum length of 2.500 bp were compiled into a FASTA catalog (-m 2,500 -nozip). Processed read data from each of the 24 samples were mapped to this catalog using Bowtie2 (version 2.3.4.3) (Langmead and Salzberg, 2012) and Samtools (version 1.9) (Li et al., 2009), with read depth being calculated using the MetaBAT2 script jgi_summarize_bam_contig_depths; default settings). We then ran VAMB (version 3.0.2) on the catalog and read depth data using default parameters except for minimum total sequence length set at 200 kb (-o C as the sample separator and -minfasta 200,000).

#### Identification of 16S SSU-rRNA Genes From Metagenome Assemblies

All contigs from the each of the 24 single assemblies and those from the coassembly contigs were analyzed for the presence of the 16S SSU-rRNA gene. Contigs was indexed with Samtools faidx (default settings) (Li et al., 2009) and analyzed with Barrnap (https://github.com/tseemann/barrnap; version 0.9), both running default settings. Annotation as full length or partial sequence was made using the –lencutoff flag, which was set to 0.8. All detected16S SSU-rRNA sequence were then annotated against SILVA version 138.1 (Quast et al., 2013) with the SINA (version 1.7.1) (Pruesse et al., 2012) with a minimum similarity of 0.95. Sets of 16S SSU-rRNA sequences were clustered using CD-HIT (Fu et al., 2012) using default options except for parameters below: sequence identity cutoff (-s) of 0.95 and minimal length similarity (fraction) of 0.6.

#### Genome Quality Estimates

Genome quality estimation of all all bins obtained from all four different pipelines (individual sample assemblies, single-BAM co-assembly, multi-BAM co-assembly and VAMB) was performed by running the CheckM (version 1.0.13) (Parks et al., 2015) lineage_wf workflow using default parameters (except -t 20 -x fa or -t 20 -x fna for VAMB bins). The output was then tabulated with the CheckM qa command using 20 threads (-t 20). MAG quality was then classified using the MIMAG criteria (Bowers et al., 2017) with modifications as follows: 1) MAGs with CheckM completeness (*C*_*p*_) and CheckM contamination (*C*_*n*_) values >90 and <5, respectively, were classified as candidates for being high quality (pHQ) genomes bins; 2) MAGs with *C*_*p*_ ≥ 50 and *C*_*n*_ < 10 were categorized as being of putatively medium quality (MQ); 3) MAGs with *C*_*p*_ <50 and *C*_*n*_ <10 were classified as candidate low quality (LQ) and 4) MAGs that did not fall into any of the above three categories were unclassified (UC). The N50 value (*N*_50_) for each MAG was calculated using QUAST version 5.0.0 (Gurevich et al., 2013) with flags -mgm -rna-finding -min-contig 1 -max-ref-number 0. for each MAG, we computed an overall (univariate) quality statistic, *Q*_*d*_ as defined by within the dRep workflow (Olm et al., 2017), defined as $Q_{d}=C_{p}-5C_{n}+\frac{C_{n}S_{h}}{100}+0.5\log N_{50}$. MAGs defined as pHQ under the MIMAG criteria were further screened for the presence of tRNAs (minimum of 18) and a complete rRNA operon (defined as the presence of at least one copy of each of the 5, 16, and 23S SSU-rRNA genes, irrespective of whether they were harbored on a single contig or not), and if present were denoted as *high quality* (HQ) MAGs.

#### Genome De-replication Procedures

We identified putative sets of cognate genomes using the dRep (version 2.2.3) (Olm et al., 2017) compare workflow executed with default settings with 20 threads (-p 20). Four dereplication analyses were performed; 1) dereplication of the complete set of MAGs; 2) de-replication of the set of 9079 MAGs combining those identified in 1) with the additional MAGs recovered from using VAMB (Supplementary Table 7); 3) de-replication of the set of 142 HQ MAGs from our analyses combined with the set of 3139 MAGs available from previously published MAG analyses of activated sludge communities (see below) and 4) The entire set of MAGs from 2) combined with the 3139 MAGs from references in dRep compare workflow with the same parameters (-p 80 -S_algorithm fastANI -multiround_primary_clustering -sa 0.95 -nc 0.3) as used in a comparable recent de-replication analysis of rumen MAG catalogs (Watson, 2021).

### Taxonomic and Functional Annotation of Recovered Genomes

Taxonomic classification of the collective set of 9079 MAG sequences was performed using the GTDB-Tk (version 0.3.2) (Chaumeil et al., 2019) classify_wf workflow with default settings (-x fa -cpus 30). Prediction of tRNA and rRNA from each recovered MAG were made using Prokka (version 1.14.6) (Seemann, 2014) executed with default parameters. Predicted rRNA genes were aligned to the SILVA database (version 138.1; release dates 12/06/2020 and 30/06/2020) (Quast et al., 2013) using SINA (version 1.7.1) (Pruesse et al., 2012) with settings -S -search-min-sim 0.95-t -v -meta-fmt csv -lca-fieldstax_slv, tax_embl, tax_ltp, tax_gg, tax_rdp.

### MAG Refinement

We performed decontamination of MAG sequences using RefineM (version 0.0.24) (Parks et al., 2017). Briefly, tetranucleotide signature and coverage profiles for contigs were calculated using scaffold_stats workflow. Contigs with divergent genomic properties were identified with outliers (default settings) and removed using the filter_bins workflow. Genes were predicted using the call_genes workflow and annotated with DIAMOND (Buchfink et al., 2015) against the gtdb_r95_protein_db.2020-07-30.dmnd and gtdb_r95_taxonomy.2020-07-30.tsv databases, within the taxon_profile workflow. Contigs with divergent taxonomic assignments were then identified with taxon_filter and removed with filter_bins. After decontamination, genome quality was reanalysed using CheckM, as described above, and bins reclassified if indicated.

### Publicly Available MAG Catalogs

We obtained the following MAG sequence data from the following published studies: 1) a set of 1083 MAGs based on long read metagenome data obtained from 23 wastewater treatment plants in Denmark (Singleton et al., 2021); 2) a set of 2045 MAGs recovered from meta-analysis of Ye et al. (2020), which includes WWTP samples from several locations in China (data collected by the authors of Ye et al., 2020), Singapore (data from Law et al., 2016), Denmark (data from Munck et al., 2015), USA (data from Chu et al., 2018), Argentina (data from Ibarbalz et al., 2016), Slovenia (data from McIlroy et al., 2016) and Switzerland (data from Ju et al., 2019); 3) one MAG sequence available from NCBI submitted from the time-series metagenome survey of a full-scale activated sludge community in Argentina (Buenos Aires) (Pérez et al., 2019); and 4) ten MAG sequences available from NCBI from the metagenome survey of three conventional WWTPs in Taiwan inoculated with exogenous anammox pellets (Yang et al., 2020). We re-estimated the genome quality of all MAGs using the CheckM based approach described above.

### Data Visualization

We constructed unrooted phylograms from MAG sequence data using GTDB-Tk (version 0.3.2) based on bacterial single-copy gene sets (bac120_ms gene sets) and imported the .tree file into R using the read.tree function from ggtree package (version 2.4.2) (Yu et al., 2017) and subsequently rendered using the ggtree function. Venn diagrams were constructed using the R package VennDiagram (version 1.6.0) (Chen and Boutros, 2011).

## Data Availability Statement

The raw data and high quality MAG sequences analysed in this study are available NCBI (BioProject Accession PRJNA731554), and key data products, including metagenome assemblies and the complete set of recovered MAG sequence data, have been made publicly available on Zenodo (DOI 10.5281/zenodo.5215738). Data results relating to the composition of secondary cluster analyses from each comparative analysis are available from Supplementary Data Files 3, 4, 7, and 8.

## Author Contributions

The study was designed by YL, SW, and RW. YL and RW designed field sampling procedures, which was conducted by LL and led by YL. LL and TN performed gDNA extractions. DD-M and SS advised on gDNA extraction procedures and protocols and generated short read sequencing data. MH, KA, and RW designed and performed data analysis. The manuscript was primarily written by RW and MH with specific sections contributed by other authors. All authors contributed to data interpretation.

## Funding

This research was supported by the Singapore National Research Foundation and Ministry of Education under the Research Centre of Excellence Programme and by program grants 1102-IRIS-10-02 (to SW, RW, and SS) from the National Research Foundation (NRF). The computational work was performed in part on resources of the National Supercomputing Centre (NSCC, Singapore) supported by Project 11000984.

## Conflict of Interest

The authors declare that the research was conducted in the absence of any commercial or financial relationships that could be construed as a potential conflict of interest.

## Publisher's Note

All claims expressed in this article are solely those of the authors and do not necessarily represent those of their affiliated organizations, or those of the publisher, the editors and the reviewers. Any product that may be evaluated in this article, or claim that may be made by its manufacturer, is not guaranteed or endorsed by the publisher.

## Supplementary Material

Supplementary Table 1 Summary of sequencing read data from each of the 24 samples analysed in this study.

Supplementary Table 2 Assembly and binning statistics of each of the 24 individual assemblies and the two co-assemblies (constructed with SPAdes and MetaBAT2).

Supplementary Table 3 Summary statistics for the 2,912 secondary (non-redundant) clusters from MAGs recovered by 24 individual assemblies and two co-assemblies (constructed with SPAdes and MetaBAT2).

Supplementary Table 4 Summary data for all 7,138 MAGs recovered from 24 individual assemblies and two co-assemblies (constructed with SPAdes and MetaBAT2).

Supplementary Table 5 MAGs from 24 individual assemblies and two co-assemblies that possessed completeness of more than 90%.

Supplementary Table 6 Summary data for the MAGs recovered from VAMB workflow.

Supplementary Table 7 Secondary (non-redundant) clusters of the complete set of MAGs recovered from all four different workflows (24 individual assemblies, two co-assemblies, and VAMB).

Supplementary Table 8 Putative high quality (pHQ) MAGs recovered from all four different workflows.

## Abbreviations

AOB: ammonium oxidizing bacteria
BAM: binary alignment map (files)
DNA: deoxyribonucleic acid
EBPR: enhanced biological phosphorus removal
GAO: glycogen accumulating organism
HQ: high quality (MAG)
LQ: low quality (MAG)
MAG: metagenome assembled genome
MIMAG: Minimum Information about a Metagenome-Assembled Genome
MQ: medium quality (MAG)
NOB: nitrite oxidizing bacteria
PAO: polyphosphate-accumulating organisms
pHQ: putative high quality (MAG)
UC: unclassified (MAG)
VAMB: variational autoencoders for metagenomic binning.

## References

[B1] Almeida A. Nayfach S. Boland M. Strozzi F. Beracochea M. Shi Z. J.. (2021). A unified catalog of 204,938 reference genomes from the human gut microbiome. Nat. Biotechnol. 39, 105–114. 10.1038/s41587-020-0603-3 32690973 PMC7801254

[B2] Arkhipova I. R. (2020). Metagenome proteins and database contamination. mSphere 5, e00854-20. 10.1128/mSphere.00854-20 33148820 PMC7643828

[B3] Arumugam K. Bağc,ı C. Bessarab I. Beier S. Buchfink B. Górska A.. (2019). Annotated bacterial chromosomes from frame-shift-corrected long-read metagenomic data. Microbiome 7, 61. 10.1186/s40168-019-0665-y 30992083 PMC6469205

[B4] Arumugam K. Bessarab I. Haryono M. A. S. Liu X. Zuniga-Montanez R. E. Roy S.. (2021). Recovery of complete genomes and non-chromosomal replicons from activated sludge enrichment microbial communities with long read metagenome sequencing. npj Biofilms Microbiomes 7, 23. 10.1038/s41522-021-00196-6 33727564 PMC7966762

[B5] Bertrand D. Shaw J. Kalathiyappan M. Ng A. H. Q. Kumar M. S. Li C.. (2019). Hybrid metagenomic assembly enables high-resolution analysis of resistance determinants and mobile elements in human microbiomes. Nat. Biotechnol. 37, 937–944. 10.1038/s41587-019-0191-2 31359005

[B6] Bickhart D. M. Kolmogorov M. Tseng E. Portik D. M. Korobeynikov A. Tolstoganov I.. (2022). Generating lineage-resolved, complete metagenome-assembled genomes from complex microbial communities. Nat. Biotechnol. 40, 711–719. 10.1038/s41587-021-01130-z 34980911

[B7] Bishara A. Moss E. L. Kolmogorov M. Parada A. E. Weng Z. Sidow A.. (2018). High-quality genome sequences of uncultured microbes by assembly of read clouds. Nat. Biotechnol. 36, 1067–1075. 10.1038/nbt.4266 30320765 PMC6465186

[B8] Bowers R. M. Kyrpides N. C. Stepanauskas R. Harmon-Smith M. Doud D. Reddy T. B. K.. (2017). Minimum information about a single amplified genome (misag) and a metagenome-assembled genome (mimag) of bacteria and archaea. Nat. Biotechnol. 35, 725–731. 10.1038/nbt.3893 28787424 PMC6436528

[B9] Brown C. T. Moritz D. O'Brien M. P. Reidl F. Reiter T. Sullivan B. D. (2020). Exploring neighborhoods in large metagenome assembly graphs using spacegraphcats reveals hidden sequence diversity. Genome Biol. 21, 164. 10.1186/s13059-020-02066-4 32631445 PMC7336657

[B10] Buchfink B. Xie C. Huson D. H. (2015). Fast and sensitive protein alignment using diamond. Nat. Methods 12, 59–60. 10.1038/nmeth.3176 25402007

[B11] Cavicchioli R. Ripple W. J. Timmis K. N. Azam F. Bakken L. R. Baylis M.. (2019). Scientists' warning to humanity: microorganisms and climate change. Nat. Rev. Microbiol 17, 569–586. 10.1038/s41579-019-0222-5 31213707 PMC7136171

[B12] Chaumeil P.-A. Mussig A. J. Hugenholtz P. Parks D. H. (2019). Gtdb-tk: a toolkit to classify genomes with the genome taxonomy database. Bioinformatics 36, 1925–1927. 10.1093/bioinformatics/btz848 31730192 PMC7703759

[B13] Chen H. Boutros P. C. (2011). Venndiagram: a package for the generation of highly-customizable venn and euler diagrams in r. BMC Bioinformatics 12, 35. 10.1186/1471-2105-12-35 21269502 PMC3041657

[B14] Chen L.-X. Anantharaman K. Shaiber A. Eren A. M. Banfield J. F. (2020). Accurate and complete genomes from metagenomes. Genome Res. 30, 315–333. 10.1101/gr.258640.119 32188701 PMC7111523

[B15] Chu B. T. T. Petrovich M. L. Chaudhary A. Wright D. Murphy B. Wells G.. (2018). Metagenomics reveals the impact of wastewater treatment plants on the dispersal of microorganisms and genes in aquatic sediments. Appl. Environ. Microbiol. 84(5). 10.1128/AEM.02168-17 29269503 PMC5812944

[B16] DeMaere M. Z. Darling A. E. (2019). bin3c: exploiting hi-c sequencing data to accurately resolve metagenome-assembled genomes. Genome Biol. 20, 46. 10.1186/s13059-019-1643-1 30808380 PMC6391755

[B17] Ding J. Regev A. (2021). Deep generative model embedding of single-cell rna-seq profiles on hyperspheres and hyperbolic spaces. Nat. Commun. 12, 2554. 10.1038/s41467-021-22851-4 33953202 PMC8099904

[B18] Douglas G. M. Langille M. G. I. (2019). Current and Promising Approaches to Identify Horizontal Gene Transfer Events in Metagenomes. Genome Biol. Evol. 11, 2750–2766. 10.1093/gbe/evz184 31504488 PMC6777429

[B19] Fu L. Niu B. Zhu Z. Wu S. Li W. (2012). Cd-hit: accelerated for clustering the next-generation sequencing data. Bioinformatics 28, 3150–3152. 10.1093/bioinformatics/bts565 23060610 PMC3516142

[B20] Gurevich A. Saveliev V. Vyahhi N. Tesler G. (2013). Quast: quality assessment tool for genome assemblies. Bioinformatics 29, 1072–1075. 10.1093/bioinformatics/btt086 23422339 PMC3624806

[B21] Hamilton R. Kits K. D. Ramonovskaya V. A. Rozova O. N. Yurimoto H. Iguchi H.. (2015). Draft genomes of gammaproteobacterial methanotrophs isolated from terrestrial ecosystems. Genome Announc 3, e00515-15. 10.1128/genomeA.00515-15 26044417 PMC4457054

[B22] Hofmeyr S. Egan R. Georganas E. Copeland A. C. Riley R. Clum A.. (2020). Terabase-scale metagenome coassembly with metahipmer. Sci. Rep. 10, 10689. 10.1038/s41598-020-67416-5 32612216 PMC7329831

[B23] Hu P. Tom L. Singh A. Thomas B. C. Baker B. J. Piceno Y. M.. (2016). Genome-resolved metagenomic analysis reveals roles for candidate phyla and other microbial community members in biogeochemical transformations in oil reservoirs. MBio 7, e01669-e01615. 10.1128/mBio.01669-15 26787827 PMC4725000

[B24] Ibarbalz F. M. Orellana E. Figuerola E. L. M. Erijman L. (2016). Shotgun metagenomic profiles have a high capacity to discriminate samples of activated sludge according to wastewater type. Appl. Environ. Microbiol. 82, 5186–5196. 10.1128/AEM.00916-16 27316957 PMC4988215

[B25] Ju F. Beck K. Yin X. Maccagnan A. McArdell C. S. Singer H. P.. (2019). Wastewater treatment plant resistomes are shaped by bacterial composition, genetic exchange, and upregulated expression in the effluent microbiomes. ISME J. 13, 346–360. 10.1038/s41396-018-0277-8 30250051 PMC6331547

[B26] Kang D. D. Li F. Kirton E. Thomas A. Egan R. An H.. (2019). Metabat 2: an adaptive binning algorithm for robust and efficient genome reconstruction from metagenome assemblies. PeerJ. 7, e7359. 10.7717/peerj.7359 31388474 PMC6662567

[B27] Kieser S. Brown J. Zdobnov E. M. Trajkovski M. McCue L. A. (2020). Atlas: a snakemake workflow for assembly, annotation, and genomic binning of metagenome sequence data. BMC Bioinformatics 21, 257. 10.1186/s12859-020-03585-4 32571209 PMC7310028

[B28] Kowalchuk G. A. Stephen J. R. (2001). Ammonia-oxidizing bacteria: a model for molecular microbial ecology. Annu. Rev. Microbiol. 55, 485–529. 10.1146/annurev.micro.55.1.485 11544365

[B29] Langmead B. Salzberg S. L. (2012). Fast gapped-read alignment with bowtie 2. Nat. Methods 9, 357–359. 10.1038/nmeth.1923 22388286 PMC3322381

[B30] Law Y. Kirkegaard R. H. Cokro A. A. Liu X. Arumugam K. Xie C.. (2016). Integrative microbial community analysis reveals full-scale enhanced biological phosphorus removal under tropical conditions. Sci. Rep. 6, 25719. 10.1038/srep25719 27193869 PMC4872125

[B31] Li H. Handsaker B. Wysoker A. Fennell T. Ruan J. Homer N.. (2009). The sequence alignment/map format and samtools. Bioinformatics 25, 2078–2079. 10.1093/bioinformatics/btp352 19505943 PMC2723002

[B32] Lui L. M. Nielsen T. N. Arkin A. P. (2021). A method for achieving complete microbial genomes and improving bins from metagenomics data. PLoS Comput. Biol. 17, e1008972. 10.1371/journal.pcbi.1008972 33961626 PMC8172020

[B33] Mallawaarachchi V. Wickramarachchi A. Lin Y. (2020). GraphBin: refined binning of metagenomic contigs using assembly graphs. Bioinformatics 36, 3307–3313. 10.1093/bioinformatics/btaa180 32167528

[B34] Martin M. (2011). Cutadapt removes adapter sequences from high-throughput sequencing reads. EMBnet. J. 17, 10–12. 10.14806/ej.17.1.200

[B35] Martineau C. Villeneuve C. Mauffrey F. Villemur R. (2014). Complete genome sequence of hyphomicrobium nitrativorans strain nl23, a denitrifying bacterium isolated from biofilm of a methanol-fed denitrification system treating seawater at the montreal biodome. Genome Announc 2, e01165-13. 10.1128/genomeA.01165-13 24435868 PMC3894282

[B36] McIlroy S. J. Albertsen M. Andresen E. K. Saunders A. M. Kristiansen R. Stokholm-Bjerregaard M.. (2014). 'candidatus competibacter'-lineage genomes retrieved from metagenomes reveal functional metabolic diversity. ISME J. 8, 613–624. 10.1038/ismej.2013.162 24173461 PMC3930307

[B37] McIlroy S. J. Karst S. M. Nierychlo M. Dueholm M. S. Albertsen M. Kirkegaard R. H.. (2016). Genomic and in situ investigations of the novel uncultured chloroflexi associated with 0092 morphotype filamentous bulking in activated sludge. ISME J. 10, 2223–2234. 10.1038/ismej.2016.14 26905629 PMC4989306

[B38] McLellan S. L. Fisher J. C. Newton R. J. (2015). The microbiome of urban waters. Int. Microbiol. 18, 141–149. 10.2436/20.1501.01.244 27036741 PMC8793681

[B39] Munck C. Albertsen M. Telke A. Ellabaan M. Nielsen P. H. Sommer M. O. A. (2015). Limited dissemination of the wastewater treatment plant core resistome. Nat. Commun. 6, 8452. 10.1038/ncomms9452 26419330 PMC4598724

[B40] Nayfach S. Shi Z. J. Seshadri R. Pollard K. S. Kyrpides N. C. (2019). New insights from uncultivated genomes of the global human gut microbiome. Nature 568, 505–510. 10.1038/s41586-019-1058-x 30867587 PMC6784871

[B41] Nissen J. N. Johansen J. Allesøe R. L. Sønderby C. K. Armenteros J. J. A. Grønbech C. H.. (2021). Improved metagenome binning and assembly using deep variational autoencoders. Nat. Biotechnol. 39, 555–560. 10.1038/s41587-020-00777-4 33398153

[B42] Nurk S. Meleshko D. Korobeynikov A. Pevzner P. A. (2017). metaspades: a new versatile metagenomic assembler. Genome Res. 27, 824–834. 10.1101/gr.213959.116 28298430 PMC5411777

[B43] Olm M. R. Brown C. T. Brooks B. Banfield J. F. (2017). drep: a tool for fast and accurate genomic comparisons that enables improved genome recovery from metagenomes through de-replication. ISME J. 11, 2864–2868. 10.1038/ismej.2017.126 28742071 PMC5702732

[B44] Orakov A. Fullam A. Coelho L. P. Khedkar S. Szklarczyk D. Mende D. R.. (2021). Gunc: detection of chimerism and contamination in prokaryotic genomes. Genome Biol. 22, 178. 10.1186/s13059-021-02393-0 34120611 PMC8201837

[B45] Parks D. H. Imelfort M. Skennerton C. T. Hugenholtz P. Tyson G. W. (2015). CheckM: assessing the quality of microbial genomes recovered from isolates, single cells, and metagenomes. Genome Res. 25, 1043–1055. 10.1101/gr.186072.114 25977477 PMC4484387

[B46] Parks D. H. Rinke C. Chuvochina M. Chaumeil P.-A. Woodcroft B. J. Evans P. N.. (2017). Recovery of nearly 8,000 metagenome-assembled genomes substantially expands the tree of life. Nat. Microbiol. 2, 1533–1542. 10.1038/s41564-017-0012-7 28894102

[B47] Pasolli E. Asnicar F. Manara S. Zolfo M. Karcher N. Armanini F.. (2019). Extensive unexplored human microbiome diversity revealed by over 150,000 genomes from metagenomes spanning age, geography, and lifestyle. Cell 176, 649.e20–662.e20. 10.1016/j.cell.2019.01.001 30661755 PMC6349461

[B48] Pérez M. V. Guerrero L. D. Orellana E. Figuerola E. L. Erijman L. McGrath J. (2019). Time series genome-centric analysis unveils bacterial response to operational disturbance in activated sludge. mSystems 4, e00169-e00119. 10.1128/mSystems.00169-19 31266798 PMC6606829

[B49] Pruesse E. Peplies J. Glöckner F. O. (2012). SINA: accurate high-throughput multiple sequence alignment of ribosomal RNA genes. Bioinformatics 28, 1823–1829. 10.1093/bioinformatics/bts252 22556368 PMC3389763

[B50] Qiu G. Liu X. Saw N. M. M. T. Law Y. Zuniga-Montanez R. Thi S. S.. (2020). Metabolic traits of candidatus accumulibacter clade iif strain scelse-1 using amino acids as carbon sources for enhanced biological phosphorus removal. Environ. Sci. Technol. 54, 2448–2458. 10.1021/acs.est.9b02901 31790213

[B51] Quast C. Pruesse E. Yilmaz P. Gerken J. Schweer T. Yarza P.. (2013). The silva ribosomal rna gene database project: improved data processing and web-based tools. Nucleic Acids Res. 41, D590-D596. 10.1093/nar/gks1219 23193283 PMC3531112

[B52] Quince C. Delmont T. O. Raguideau S. Alneberg J. Darling A. E. Collins G.. (2017a). Desman: a new tool for de novo extraction of strains from metagenomes. Genome Biol. 18, 181. 10.1186/s13059-017-1309-9 28934976 PMC5607848

[B53] Quince C. Nurk S. Raguideau S. James R. Soyer O. S. Summers J. K.. (2021). STRONG: Metagenomics strain resolution on assembly graphs. Genome Biol. 22, 214. 10.1186/s13059-021-02419-7 34311761 PMC8311964

[B54] Quince C. Walker A. W. Simpson J. T. Loman N. J. Segata N. (2017b). Shotgun metagenomics, from sampling to analysis. Nat. Biotechnol. 35, 833–844. 10.1038/nbt.3935 28898207

[B55] R Core Team (2021). R: A Language and Environment for Statistical Computing. Vienna: R Foundation for Statistical Computing.

[B56] Ricaboni D. Mailhe M. Khelaifia S. Raoult D. Million M. (2016). Romboutsia timonensis, a new species isolated from human gut. New Microbes New Infect. 12, 6–7. 10.1016/j.nmni.2016.04.001 27200178 PMC4864248

[B57] Sangwan N. Xia F. Gilbert J. A. (2016). Recovering complete and draft population genomes from metagenome datasets. Microbiome 4, 8. 10.1186/s40168-016-0154-5 26951112 PMC4782286

[B58] Seemann T. (2014). Prokka: rapid prokaryotic genome annotation. Bioinformatics 30, 2068–2069. 10.1093/bioinformatics/btu153 24642063

[B59] Singleton C. M. Petriglieri F. Kristensen J. M. Kirkegaard R. H. Michaelsen T. Y. Andersen M. H.. (2021). Connecting structure to function with the recovery of over 1000 high-quality metagenome-assembled genomes from activated sludge using long-read sequencing. Nat. Commun. 12, 2009. 10.1038/s41467-021-22203-2 33790294 PMC8012365

[B60] Skennerton C. T. Barr J. J. Slater F. R. Bond P. L. Tyson G. W. (2015). Expanding our view of genomic diversity in candidatus accumulibacter clades. Environ. Microbiol. 17, 1574–1585. 10.1111/1462-2920.12582 25088527

[B61] Soo R. M. Skennerton C. T. Sekiguchi Y. Imelfort M. Paech S. J. Dennis P. G.. (2014). An expanded genomic representation of the phylum cyanobacteria. Genome Biol Evol 6, 1031–1045. 10.1093/gbe/evu073 24709563 PMC4040986

[B62] Stewart R. D. Auffret M. D. Warr A. Walker A. W. Roehe R. Watson M. (2019). Compendium of 4,941 rumen metagenome-assembled genomes for rumen microbiome biology and enzyme discovery. Nat. Biotechnol. 37, 953–961. 10.1038/s41587-019-0202-3 31375809 PMC6785717

[B63] Stokholm-Bjerregaard M. McIlroy S. J. Nierychlo M. Karst S. M. Albertsen M. Nielsen P. H. (2017). A critical assessment of the microorganisms proposed to be important to enhanced biological phosphorus removal in full-scale wastewater treatment systems. Front. Microbiol 8, 718. 10.3389/fmicb.2017.00718 28496434 PMC5406452

[B64] Tully B. J. Graham E. D. Heidelberg J. F. (2018). The reconstruction of 2,631 draft metagenome-assembled genomes from the global oceans. Scientific Data 5, 170203. 10.1038/sdata.2017.203 29337314 PMC5769542

[B65] Uritskiy G. V. DiRuggiero J. Taylor J. (2018). Metawrap-a flexible pipeline for genome-resolved metagenomic data analysis. Microbiome 6, 158. 10.1186/s40168-018-0541-1 30219103 PMC6138922

[B66] Vicedomini R. Quince C. Darling A. E. Chikhi R. (2021). Strainberry: automated strain separation in low-complexity metagenomes using long reads. Nat. Commun. 12, 4485. 10.1038/s41467-021-24515-9 34301928 PMC8302730

[B67] Vijayan A. Vattiringal Jayadradhan R. K. Pillai D. Prasannan Geetha P. Joseph V. Isaac Sarojini B. S. (2021). Nitrospira as versatile nitrifiers: Taxonomy, ecophysiology, genome characteristics, growth, and metabolic diversity. J. Basic Microbiol. 61, 88–109. 10.1002/jobm.202000485 33448079

[B68] Vollmers J. Wiegand S. Kaster A.-K. (2017). Comparing and evaluating metagenome assembly tools from a microbiologist's perspective - not only size matters! PLoS ONE 12, e0169662. 10.1371/journal.pone.0169662 28099457 PMC5242441

[B69] Watson M. (2021). New insights from 33,813 publicly available metagenome-assembled-genomes (mags) assembled from the rumen microbiome. bioRxiv. 10.1101/2021.04.02.438222

[B70] Wu L. Ning D. Zhang B. Li Y. Zhang P. Shan X.. (2019). Global diversity and biogeography of bacterial communities in wastewater treatment plants. Nat. Microbiol. 4, 1183–1195. 10.1038/s41564-019-0426-5 31086312

[B71] Yang Y. Pan J. Zhou Z. Wu J. Liu Y. Lin J.-G.. (2020). Complex microbial nitrogen-cycling networks in three distinct anammox-inoculated wastewater treatment systems. Water Res. 168, 115142. 10.1016/j.watres.2019.115142 31605831

[B72] Ye L. Mei R. Liu W.-T. Ren H. Zhang X.-X. (2020). Machine learning-aided analyses of thousands of draft genomes reveal specific features of activated sludge processes. Microbiome 8, 16. 10.1186/s40168-020-0794-3 32046778 PMC7014675

[B73] Yu G. Smith D. K. Zhu H. Guan Y. Lam T. T.-Y. (2017). ggtree: an r package for visualization and annotation of phylogenetic trees with their covariates and other associated data. Methods Ecol. Evol. 8, 28–36. 10.1111/2041-210X.12628

